# Research Advances on Human-Eye-Sensitive Long Persistent Luminescence Materials

**DOI:** 10.3389/fchem.2021.654347

**Published:** 2021-05-07

**Authors:** Yuhua Wang, Haijie Guo

**Affiliations:** ^1^Key Laboratory for Special Function Materials and Structure Design of the Ministry of Education, Lanzhou University, Lanzhou, China; ^2^Department of Materials Science, School of Physical Science and Technology, Lanzhou University, Lanzhou, China

**Keywords:** persistent luminescence, Eu^2+^ ions, sensitivity to human eyes, muticolor, energy transfer

## Abstract

Based on the actual application requirements of multicolor long persistent luminescence (LPL) materials, we highlight the recent developments in the last decade on human-eye-sensitive LPL materials and try to make a full list of known LPL compounds possessing wavelengths of 400–600 nm and a duration time longer than 10 h (>0.32 mcd/m^2^); these are more sensitive to the human eye's night vision and can be used throughout the night. We further emphasize our group research of novel LPL materials and the regulation of LPL color to enable a full palette. In the end, we try to summarize the challenges and perspectives of LPL materials for potential research directions based on our limited understandings. This review could offer new enlightenment for further exploration of new LPL materials in the visible light range and related applications.

## Introduction

Long persistent luminescence (LPL) materials are defined as energy-saving and environmentally friendly materials that show persistent luminescence from seconds to hours after turning off the excitation sources, such as UV, visible light, X-ray, and sunlight (Zhuang et al., 2016; Li Y. et al., 2017; Xu et al., 2018; Xiong et al., 2019). Up to now, the most efficient visible light LPL materials remain blue CaAl_2_O_4_: Eu^2+^, Nd^3+^ and green SrAl_2_O_4_: Eu^2+^, Dy^3+^ (Katsumata et al., 1997, 1998; Hölsä et al., 2001), which both struggle to achieve multicolor and extensive applications. On this basis, it is the right time to provide this review to further stimulate and develop novel visible-light-emitting LPL materials and open up new applications.

As we all know, visible light can be observed and distinguished by human eyes, but the sensitivity of human eyes toward different colors is quite different. In addition, the sensitivity of the human eye also varies according to the ambient brightness (Hölsä, 2009; Poelman and Smet, 2010; Brito et al., 2012). Human eye sensitivity functions have a peak of 683 lm/W at 555 nm in photopic vision and 1,700 lm/W at 507 nm in scotopic vision (Poelman et al., 2009). That is, in scotopic vision (LPL material application conditions), human eyes are more sensitive to ~400–600 nm light. Therefore, the development of materials in this wavelength range is very important for practical applications, especially in night vision. At the same time, compared with reviews concerning LPL materials classified by emission wavelength (UV, Red, and NIR), matrices, and activators, common features, including the history, mechanism, and application of LPL materials, have been reported on repeatedly and will not be elaborated on here (Eeckhout et al., 2010, 2013; Smet et al., 2014; Zhuang et al., 2014; Li et al., 2016; Xiong and Peng, 2019; Xu and Tanabe, 2019). In this review, we mainly focused on the following three aspects: (1) the recent development of novel LPL materials with wavelengths of 400–600 nm; (2) the approach of regulating the LPL color to achieve multicolor and discoloration on the basis of our own work; (3) the challenges and perspectives of LPL material for potential research directions. This work may be useful for the exploration of human-eye-sensitive LPL materials and the further optimization of LPL performance.

## Advances in LPL Materials Sensitive to Human Eyes

As far as we know, reviews concerning the development of LPL materials with wavelengths of 400–600 nm, which play important roles in practical application, have rarely been made. In this regard, we try to make a full list of known LPL materials, possessing 400–600 nm wavelengths and a decay time longer than 10 h (>0.32 mcd/m^2^), which can be used throughout the whole night, as shown in Table 1 (Gong et al., 2009; Jiang et al., 2013; Luitel et al., 2013; Zeng et al., 2013; Yu et al., 2014; Cheng et al., 2015; Guo et al., 2015, 2017, 2019; Wang et al., 2015; Zou et al., 2015; Jia et al., 2016; Wang S. et al., 2017; Wang W. et al., 2017; Dou et al., 2018; Feng et al., 2019). Unsurprisingly, only a small amount of ions, such as Bi^3+^ and Mn^2+^, act as activators of LPL materials. This is particularly the case for Bi^3+^ ions, which have gradually attracted people's attention in recent years. As typical examples, Wang et al. synthesized NaLuGeO_4_: Bi^3+^, Eu^3+^ LPL material in 2017 (Wang W. et al., 2017). At 316 nm, a wide packet emission occurs in the 330–500 nm range, peaking at 400 nm, and this is ascribed to ^3^P_1,0_ → ^1^S_0_ transitions of Bi^3+^ ions. The LPL can still be detected 63 hours after the light source is turned off. Xu et al. first realized the yellow super LPL in 2017 through Bi^3+^ ion doping in CaGa_2_O_4_ (Wang S. et al., 2017). Excited by 337 nm, the yellow emission, with a peak value of 584 nm, was detected. After excitation at 254 nm and 365 nm for 15 min, the LPL decay time exceeded 24 h. Bi^3+^ ions not only act as luminescence centers but also as trap centers to effectively improve electron transfer rates. In 2018, Li et al. reported that the emission range of CaZnGe_2_O_6_: Bi^3+^ is 330–500 nm, the peak value is 468 nm, and the LPL decay time can last for more than 12 h after 254 nm light excitation for 30 min (Dou et al., 2018). Furthermore, except for Bi^3+^ ions, Mn^2+^ ions also have been recently reported to act as activators in some LPL phosphors (Lei et al., 2006; Che et al., 2008; Takahashi et al., 2011; Xu et al., 2013; Zou et al., 2015; Zhou et al., 2018). Unfortunately, the overall LPL performance is inferior. Among them, ZnSiO_4_: Mn^2+^, Yb^3+^, reported by Zhang et al., has a glorious green LPL performance, peaking at 523 nm (Zou et al., 2015). After 15 min of exposure to 254 nm light, the LPL duration time can last ~30 h (0.32 mcd/m^2^). Although the past decade or so has witnessed significant advances in LPL materials with wavelengths in the range of 400–600 nm, the pattern of Eu^2+^ ions acted as activator ions and lanthanide rare-earth ions serving as trapping centers or to produce defect-related trapping center is still the most effective for most excellent LPL materials. On these grounds, we mainly introduce the research of our group on ultra LPL materials doped with Eu^2+^ ions in the next chapter.

**Table 1:** Recently reported LPL materials possessing 400–600 nm wavelengths and longer than 10 h duration time (0.32 mcd/m^2^).

**Emission peak (nm)**	**Materials**	**Activator**	**Co-dopant**	**Duration time (h)**	**Initial intensity (mcd/m^**2**^)**	**Excitation wavelength (nm)**	**References**
400	NaLuGeO_4_	Bi^3+^	Eu^3+^	~63	–	–	Wang W. et al., 2017
468	CaZnGe_2_O_6_	Bi^3+^	-	>12	–	254	Dou et al., 2018
473	Ba_5_Si_8_O_21_	Eu^2+^	Dy^3+^	>16	>100	365	Wang et al., 2015
478	BaZrSi_3_O_9_	Eu^2+^	Pr^3+^	>15	238	254	Guo et al., 2017
494	Sr_4_Al_14_O_25_	Eu^2+^	Dy^3+^	>20	–	–	Luitel et al., 2013
501	Ba_2_Zr_2_Si_3_O_12_	Eu^2+^	Nd^3+^	>25	155	254	Guo et al., 2019
514	BaSi_2_O_5_	Eu^2+^	Pr^3+^	~38	–	254	Feng et al., 2019
523	Zn_2_SiO_4_	Mn^2+^	Yb^3+^	~30	–	254	Zou et al., 2015
535	Ca_2_MgSi_2_O_7_	Eu^2+^	Ce^3+^	~20	–	Artificial daylight	Gong et al., 2009
553	Ca_6_BaP_4_O_17_	Eu^2+^	Ho^3+^	~47	130	254	Guo et al., 2015
560	BaSiO_3_	Eu^2+^	Nd^3+^, Tm^3+^	~10	–	Sunlight	Jia et al., 2016
567	Li_2_SrSiO_4_	Eu^2+^	Dy^3+^	>15	–	254	Cheng et al., 2015
574	Sr_3_SiO_5_	Eu^2+^	Lu^3+^	~20	–	365	Yu et al., 2014
580	Ca_2_BO_3_Cl	Eu^2+^	Dy^3+^	~48	>100	254	Zeng et al., 2013
580	Ca_2_ZnSi_2_O_7_	Eu^2+^	Dy^3+^	~12	–	460	Jiang et al., 2013
584	CaGa_2_O_4_	Bi^3+^	–	~24	–	254	Wang S. et al., 2017

## The Approach to Achieve Multicolor LPL Based on Our Own Work

### Exploiting Novel LPL Materials With Rare Colors

**Figure 1:**
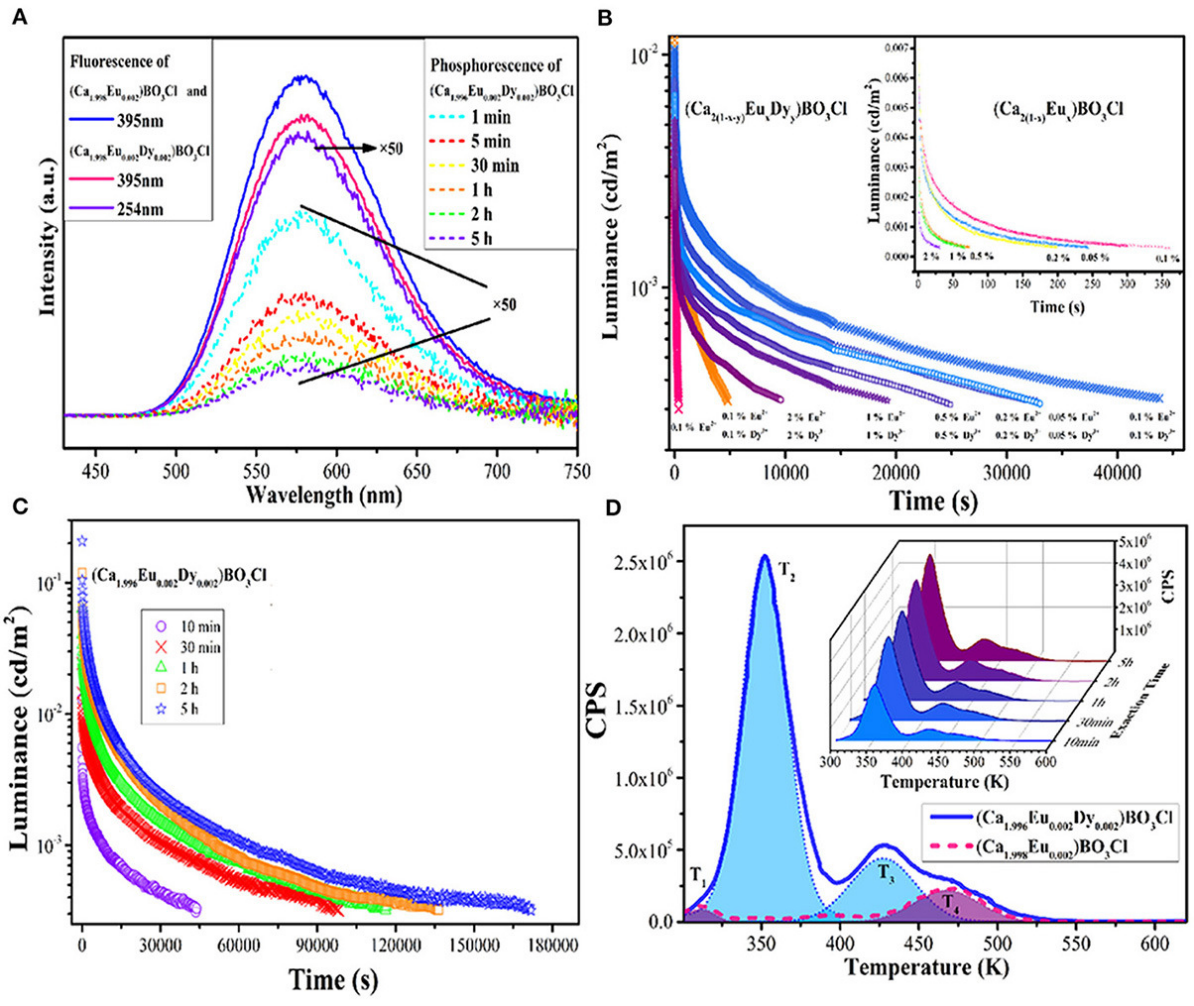
**(A)** PL spectra of Ca_2_BO_3_Cl: 0.002Eu^2+^ and Ca_2_BO_3_Cl: 0.002Eu^2+^, 0.002Dy^3+^ and LPL spectra of Ca_2_BO_3_Cl: 0.002Eu^2+^, 0.002Dy^3+^. **(B)** LPL decay curves of Ca_2_BO_3_Cl: xEu^2+^, yDy^3+^ and Ca_2_BO_3_Cl: xEu^2+^ samples (inset). **(C)** LPL decay curves of Ca_2_BO_3_Cl: 0.002Eu^2+^, 0.002Dy^3+^ excited at different times. **(D)** TL curves of Ca_2_BO_3_Cl: 0.002Eu^2+^, 0.002Dy^3+^ and Ca_2_BO_3_Cl: 0.002Eu^2+^ excited for 10 min and TL curves of Ca_2_BO_3_Cl: 0.002Eu^2+^, 0.002Dy^3+^ excited for different times (inset). Reproduced by permission of The Royal Society of Chemistry.

We know that yellow is often used to warn of danger or draw attention, such as yellow lights on traffic signs, large construction machinery, raincoats, etc. If the material itself continues to emit yellow light in dark conditions, this will further improve security and reliability. In addition, to date, only scattered yellow LPL materials have been reported, and the main reason is that the crystal field strength should be strong enough to reduce the 5d energy level of Eu^2+^ and thus produce yellow luminescence (Lakshminarasimhan and Varadaraju, 2008; Sun et al., 2008; Li et al., 2013, 2014). Here, we mainly present two excellent yellow materials: Ca_2_BO_3_Cl: Eu^2+^, Dy^3+^ and Ca_6_BaP_4_O_17_: Eu^2+^, Ho^3+^. These have been exploited by our research group (Zeng et al., 2013; Guo et al., 2015). First of all, in the Ca_2_BO_3_Cl borate matrix, the photo-luminescence (PL) and LPL spectra of Ca_2_BO_3_Cl: Eu^2+^, Dy^3+^ have the same profile and both present an asymmetric wide emission band at 580 nm, indicating that Eu^2+^ ions occupying both Ca^2+^ lattice sites can act as a luminescent center in PL and LPL processes (Figure 1). For the representative sample of Ca_2_BO_3_Cl: 0.002Eu^2+^, 0.002Dy^3+^, after 10 min excitation of 254 nm and 365 nm lights, the initial LPL intensity can achieve 0.01 cd/m^2^ and its LPL can sustain more than 12 h above 0.32 mcd/m^2^. As the excitation time is further extended from 10 min to 5 h, the thermoluminescence (TL) intensity, LPL initial brightness, and decay time are significantly improved and prolonged. When the excitation time is up to 5 h, the charging procedure approaches saturation, and the LPL decay time reaches 48 h. This large energy storage capacity also provide benefits of optical storage and other potential applications as well (Zhuang et al., 2018).

**Figure 2:**
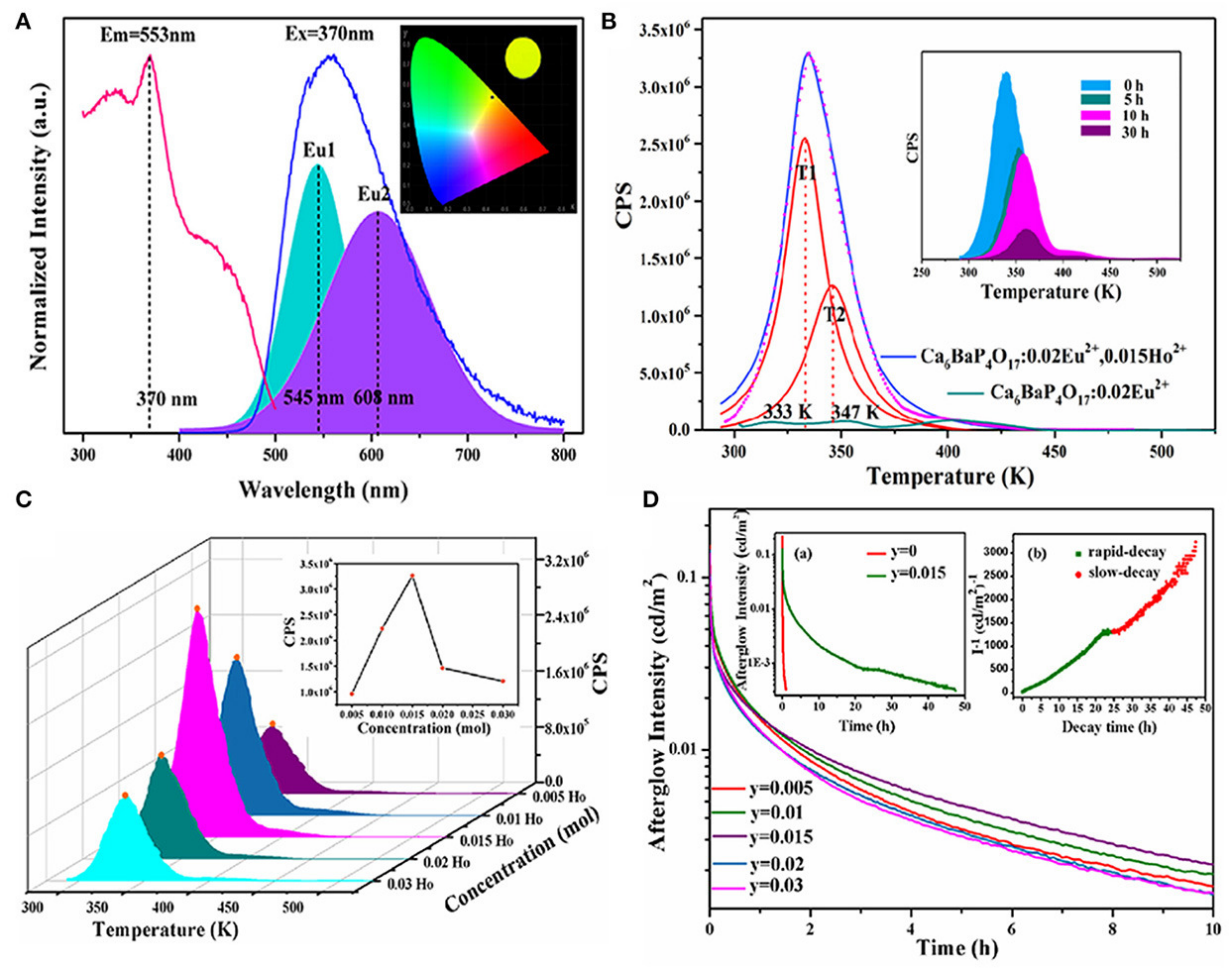
**(A)** PLE and PL spectra of Ca_6_BaP_4_O_17_: 0.02Eu^2+^, 0.015Ho^3+^. Inset: CIE chromaticity diagram for Ca_6_BaP_4_O_17_: 0.02Eu^2+^, 0.015Ho^3+^ excited by 370 nm. **(B)** TL curves of Ca_6_BaP_4_O_17_: 0.02Eu^2+^, 0.015Ho^3+^ and Ca_6_BaP_4_O_17_: 0.02Eu^2+^ excited for 2 min. Inset: TL curves of Ca_6_BaP_4_O_17_: 0.02Eu^2+^, 0.015Ho^3+^ excited for 2 min and placed in a dark room for different times. **(C)** TL curves of Ca_6_BaP_4_O_17_: 0.02Eu^2+^, yHo^3+^ (0.005 ≤ y ≤ 0.03) excited for 2 min. **(D)** LPL decay curves of Ca_6_BaP_4_O_17_: 0.02Eu^2+^, yHo^3+^ (0.005 ≤ y ≤ 0.03). Inset show the LPL decay curves of Ca_6_BaP_4_O_17_: 0.02Eu^2+^ and Ca_6_BaP_4_O_17_: 0.02Eu^2+^, 0.015Ho^3+^ and the function of reciprocal afterglow intensity (*I*^−1^) vs. time (*t*) of Ca_6_BaP_4_O_17_: 0.02Eu^2+^, 0.015Ho^3+^ excited for 15 min. Reproduced by permission of The rOyal Society of Chemistry.

Furthermore, as shown in Figure 2, in the Ca_6_BaP_4_O_17_ phosphate matrix, both PL and LPL spectra of Ca_6_BaP_4_O_17_: 0.02Eu^2+^, 0.015Ho^3+^ only have an asymmetric broad emission band peaking at 553 nm, resulting in a bright yellow PL and LPL. Incorporation of Ho^3+^ ions can largely extend the TL characteristics and evidently elevate the LPL performance of Ca_6_BaP_4_O_17_: Eu^2+^, Ho^3+^. For Ca_6_BaP_4_O_17_: 0.02Eu^2+^, we just observe three very weak TL peaks, which indicate that the electron concentration trapped at the intrinsic defects is very low. Whereas, codoping Ho^3+^ ions makes the TL peak at 335 K greatly enhanced and largely improves the defect levels. After 15 min excitation of 254 nm and 365 nm lights, for Ca_6_BaP_4_O_17_: 0.02Eu^2+^, 0.015Ho^3+^, the initial LPL brightness can reach about 0.13 cd/m^2^ and LPL can last more than 47 h above 0.32 mcd/m^2^.

**Figure 3:**
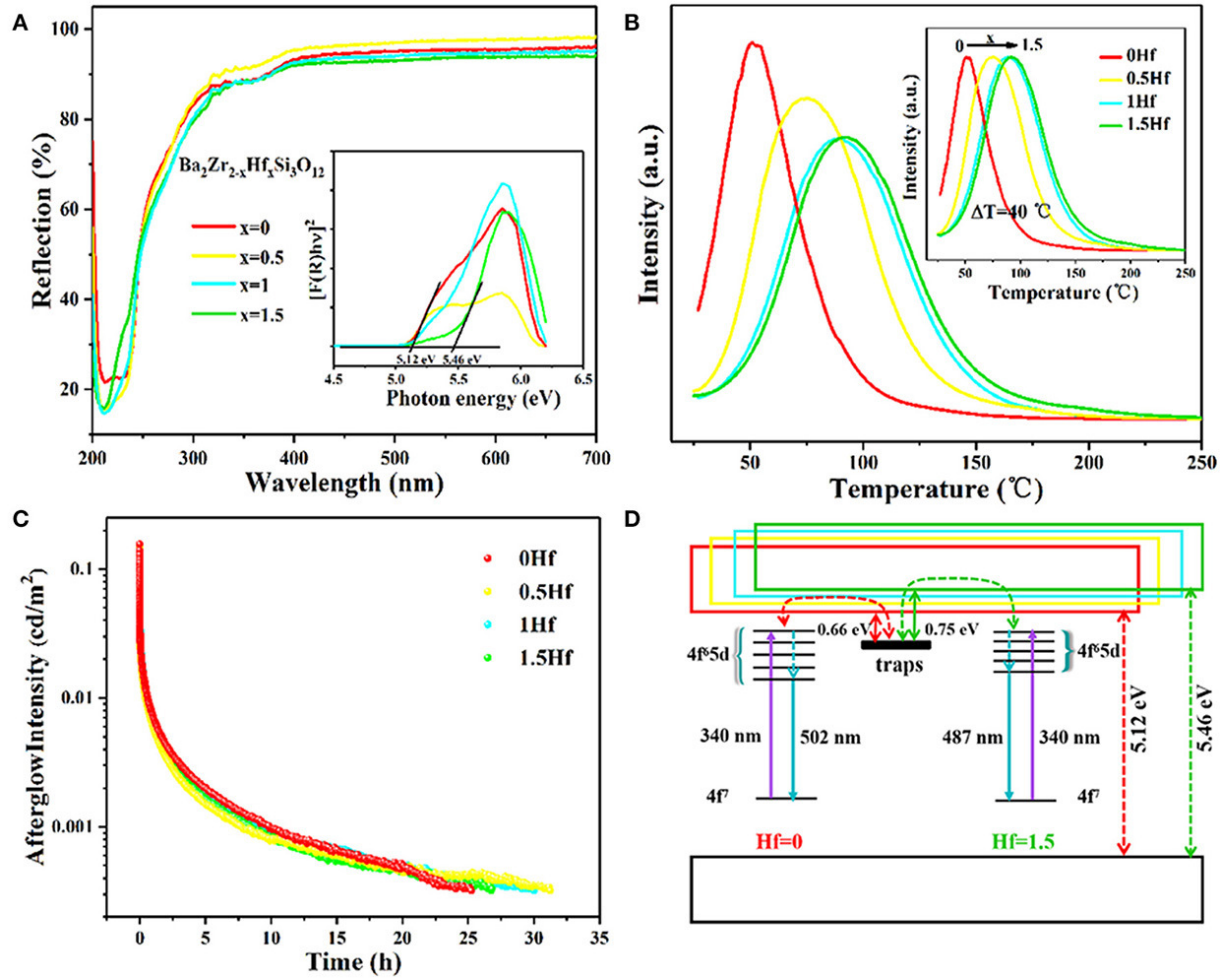
**(A)** Diffuse reflectance spectra and Tauc plots (inset) of Ba_2_Zr_2−x_Hf_x_Si_3_O_12_ (*x* = 0, 0.5, 1, 1.5) samples. **(B)** The TL spectra, **(C)** LPL decay curves, and **(D)** Schematic energy level diagrams of Ba_2_Zr_2−x_Hf_x_Si_3_O_12_: 0.01Eu^2+^, 0.01Nd^3+^ (*x* = 0, 0.5, 1, 1.5). Reprinted with permission from Guo et al. (2019). Copyright (2019) American Chemical Society.

Looking from the other side, in the dark environment (10^−6^~10^−2^ cd/m^2^), the maximum visual sensitivity of the human eye is 507 nm. That is to say, the human eye is most sensitive to cyan light in the dark. Because of this, our research team was striving to exploit cyan LPL materials. We first identified Ba_2_Zr_2_Si_3_O_12_: Eu^2+^, Nd^3+^ cyan LPL material (Guo et al., 2019). Under 340 nm excitation, Ba_2_Zr_2_Si_3_O_12_: 0.01Eu^2+^, 0.01Nd^3+^ possess a broad asymmetric cyan PL band at ~501 nm. The LPL initial intensity is 155.5 mcd/m^2^, and the LPL duration time is near 25 h before decaying to 0.32 mcd/m^2^. On this basis, we tried to adjust the bandgap of Ba_2_Zr_2_Si_3_O_12_ by replacing Zr^4+^ with Hf^4+^ ions due to the conduction band (CB) bottom of Ba_2_Zr_2_Si_3_O_12_ is mainly composed of Zr 4d electron states, and thus the energy gap of the excited state of Eu^2+^ ions and trap center relative to CB can also be regulated. As a result, with the increase of Hf^4+^ concentration, the bottom of the CB moves upward, making the bandgap vary from 4.288 to 4.301 eV and the TL peak shift monotonically from 51 to 91°C, corresponding to the trap depth moves from 0.66 to 0.76 eV, as presented in Figure 3. For the optimal sample (*x* = 0.5), the initial brightness of LPL can reach 131.3 mcd/m^2^, and the LPL decay time can last for 31 h.

**Figure 4:**
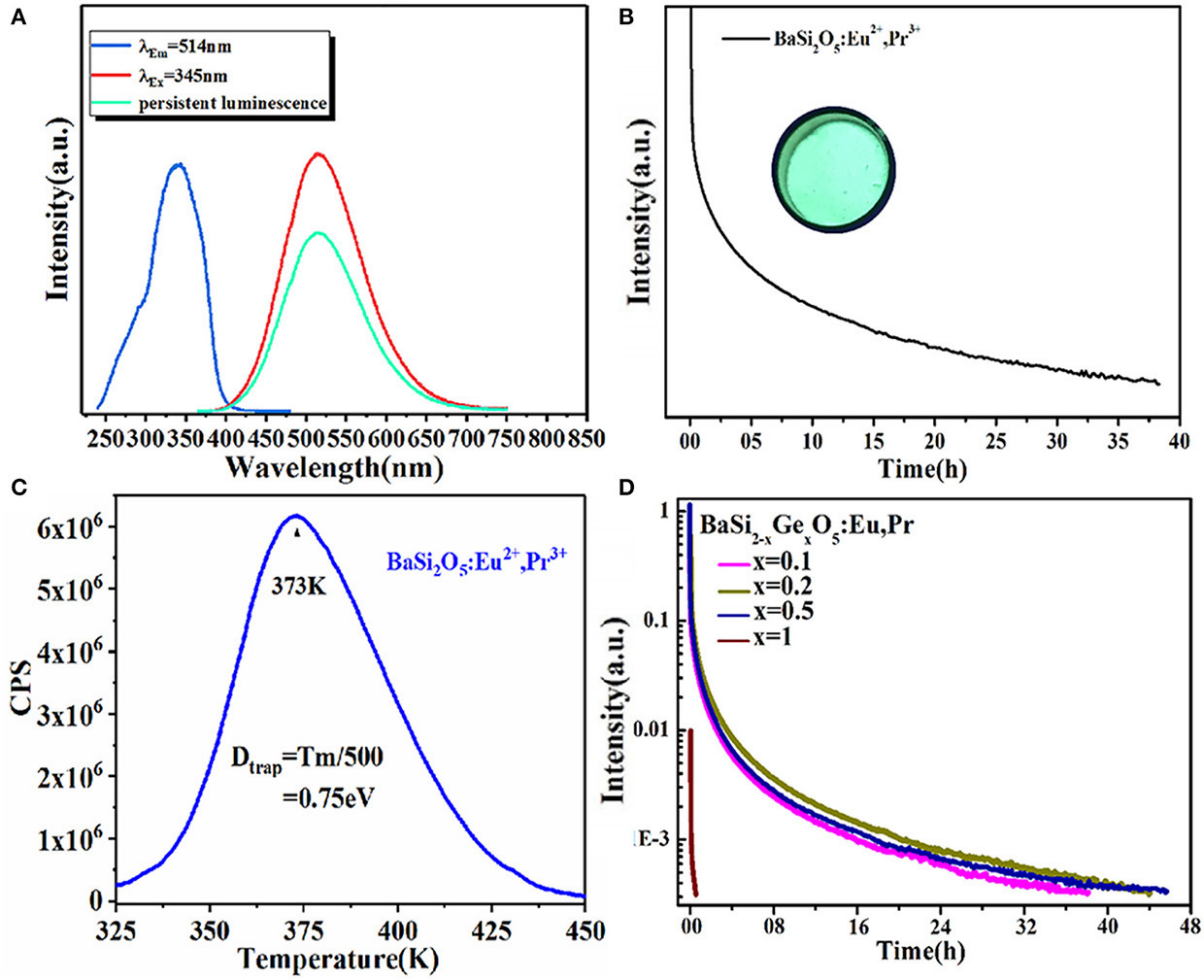
**(A)** The PLE, PL and LPL spectra of BaSi_2_O_5_: 0.008Eu^2+^, 0.01Pr^3+^. **(B)** LPL decay curve of BaSi_2_O_5_: 0.008Eu^2+^, 0.01Pr^3+^. Inset: the digital photo under 254 nm irradiation. **(C)** TL spectra of BaSi_2_O_5_: 0.008Eu^2+^, 0.01Pr^3+^. **(D)** LPL decay curves of BaSi_2−x_Ge_x_O_5_: 0.008Eu^2+^, 0.01Pr^3+^ (0 ≤ x ≤ 1). Reprinted with permission from Feng et al. (2019). Copyright (2019) American Chemical Society.

Moreover, a series of (Ba, Li) (Si, Ge, P)_2_O_5_: Eu^2+^, Pr^3+^ cyan LPL materials were designed by adopting a solid solution strategy (Feng et al., 2019). After 10 min of excitation at 254 nm, BaSi_2_O_5_: 0.008Eu^2+^, 0.01Pr^3+^ presents a strong cyan LPL located at 514 nm and the LPL duration time reaches about 38 h, which is ascribed to the increased TL intensity at 350 K by codoping Pr^3+^ ions and thus generating a number of traps suitable for LPL at room temperature (Figure 4). Furthermore, by means of solid solution, the LPL decay time of (Ba, Li) (Si, Ge, P)_2_O_5_: Eu^2+^, Pr^3+^ can be prolonged from 38 to 56 h.

For the abovelisted yellow and cyan LPL materials, the LPL properties have been greatly improved. The only disadvantage, same with the current common problem of LPL materials, is that they cannot be effectively excited by sunlight. This situation limits the wide application utilizing solar energy outdoors. However, emergencies, energy conservation, and multi-color requirements can still be realized through combining with UV chip and short-term charging.

### Realizing Energy Transfer

**Figure 5:**
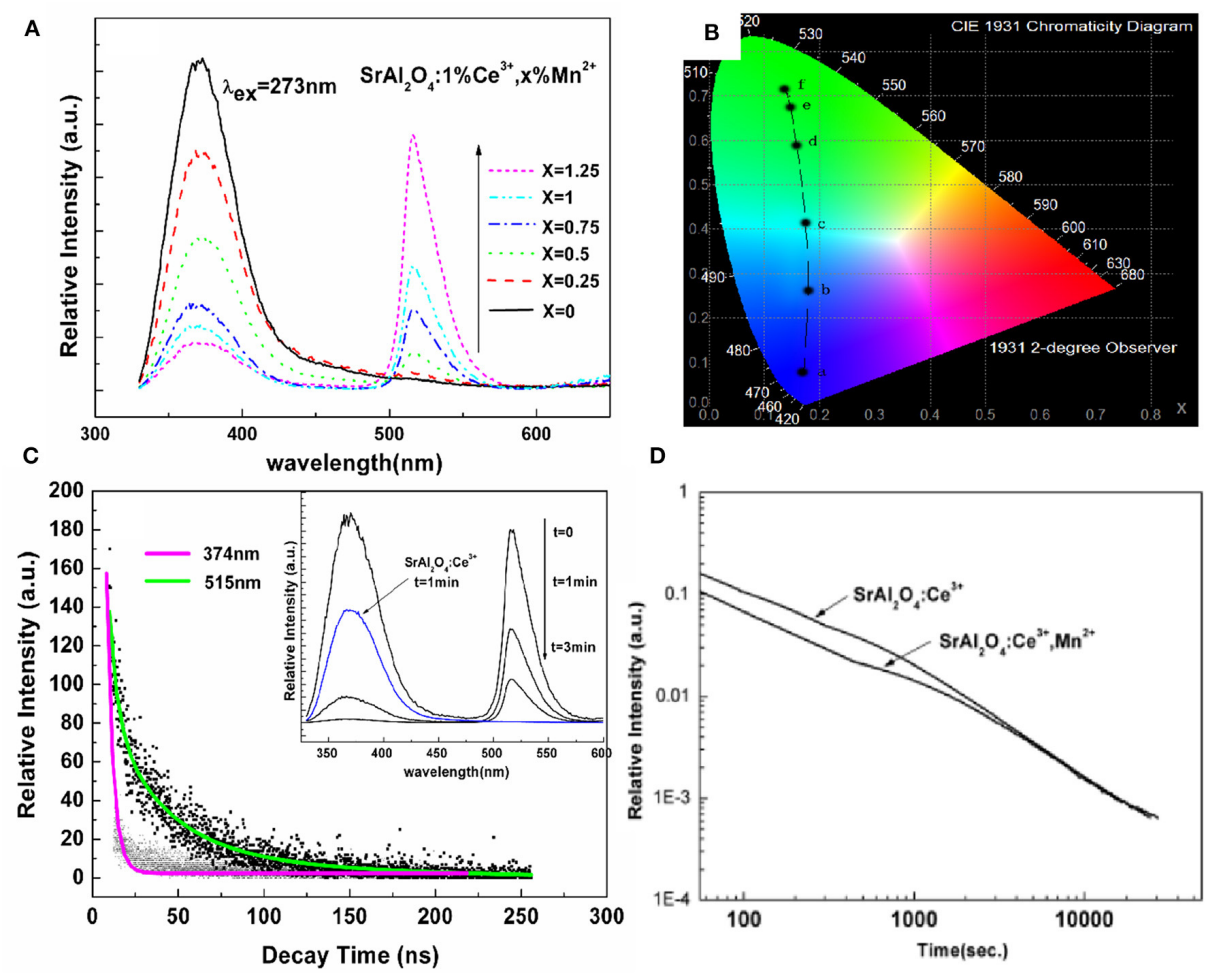
**(A)** Emission spectra, and **(B)** CIE chromaticity diagram for SrAl_2_O_4_: 1% Ce^3+^, x% Mn^2+^ (*x* = 0, 0.25, 0.5, 0.75, 1, and 1.25) excited by 273 nm. **(C)** Lifetime decay curves of 370 and 515 nm emissions in SrAl_2_O_4_: 1%Ce^3+^, 0.75%Mn^2+^ phosphor. Inset: the LPL spectra of SrAl_2_O_4_: 1%Ce^3+^, 0.75%Mn^2+^ after switching off the excitation source at different times. **(D)** LPL decay curves of SrAl_2_O_4_: 1%Ce^3+^ and SrAl_2_O_4_: 1%Ce^3+^, 0.75%Mn^2+^ after 10 min excitation of artificial light. Reprinted with permission from Xu et al. (2011). Copyright (2010) The American Ceramic Society.

On the basis of the above work, we can easily find that it is difficult to realize the diversified LPL colors in the same material. In particular, for Eu^2+^ ions, the LPL color in most matrices is blue-green. In a matrix, when the emission spectrum of one luminescent center overlapped with the excitation spectrum of another luminescent center, it was possible to realize non-radiative energy transfer between two luminescent centers, such as Eu^2+^ → Mn^2+^, Ce^3+^ → Mn^2+^, and Ce^3+^ → Tb^3+^ (Jia et al., 2002; Wang et al., 2003; Xu et al., 2009a, 2011; Dai, 2014). Multicolor LPL could be achieved by the energy transfer between two luminescent centers based on the existence of a large number of trap centers. As shown in Figure 5, in SrAl_2_O_4_: Ce^3+^ blue LPL material (Xu et al., 2011), with the increase of Mn^2+^ ions concentration, the emission of Mn^2+^ ions located at 515 nm increases, and the emission band of Ce^3+^ ions at 374 nm, decreases, proving that there is an effective energy transfer from Ce^3+^ to Mn^2+^ ions. We improved the energy transfer efficiency by adjusting the concentration of Mn^2+^ ions and realized the adjustable PL color from blue light to green light. When the excitation source is removed, the energy transfer from Ce^3+^ to Mn^2+^ ions continues. However, due to the energy loss in the energy transfer process and the different decay rates of the two ions, it is difficult to ensure the uniformity of LPL color. Among them, the inevitable energy loss in the LPL energy transfer process is also one of the main reasons for the initial LPL brightness reduction.

**Figure 6:**
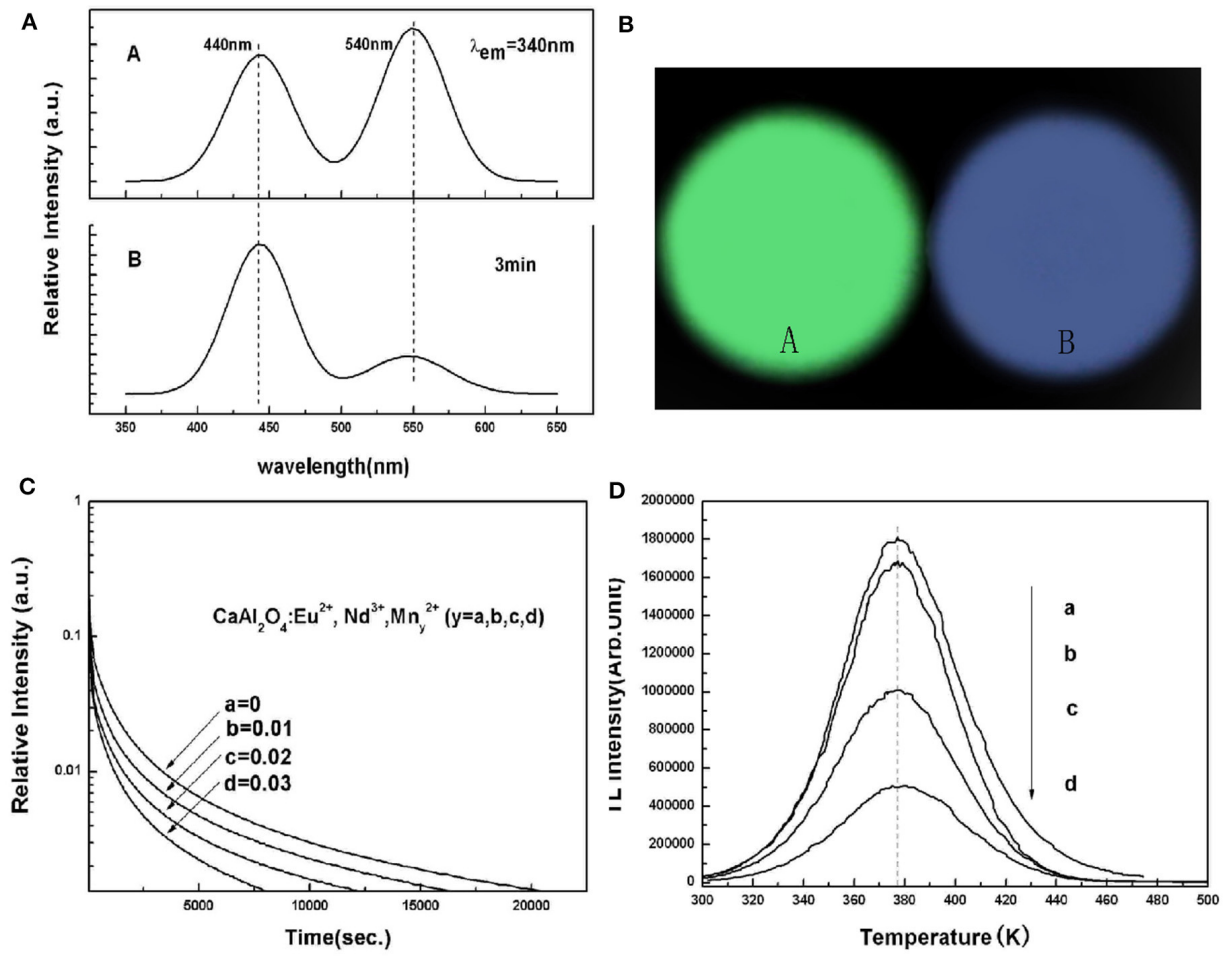
**(A)** PL and LPL spectra of CaAl_2_O_4_: xEu^2+^, 2xNd^3+^, yMn^2+^ (*x* = 0.00125, *y* = 0.03). **(B)** Photographs of PL and LPL of CaAl_2_O_4_: xEu^2+^, 2xNd^3+^, yMn^2+^ (*x* = 0.00125, *y* = 0.03). **(C)** LPL decay curves, and **(D)** TL curves of Ca_1−3x−y_Al_2_O_4_: xEu^2+^, 2xNd^3+^, yMn^2+^ (*x* = 0.00125, 0 ≤ y ≤ 0.03). Rights managed by AIP Publishing.

In addition, in CaAl_2_O_4_:Eu^2+^, Nd^3+^ blue LPL material, we realized the energy transfer between Eu^2+^ and Mn^2+^ ions through codoping different Mn^2+^ concentrations, and achieved the regulation of PL color from blue to green, as shown in Figure 6 (Xu et al., 2009a). Unfortunately, it can be seen from the LPL spectrum that the energy transfer efficiency of Eu^2+^ and Mn^2+^ ions is lower in the LPL decay process so that the LPL color is still blue. Even worse, the incorporation of Mn^2+^ ions leads to poor LPL performances. There are two main reasons. First, the incorporation of Mn^2+^ ions significantly reduced the concentration of the effective trap center, which can be observed from the TL spectra. Second, in the LPL decay process, the energy transfer efficiency between Eu^2+^ and Mn^2+^ ions is so low leading to inevitable energy loss. Therefore, the LPL color regulation can not be easily realized through energy transfer in the same substrate, and the LPL performance can also deteriorate.

**Figure 7:**
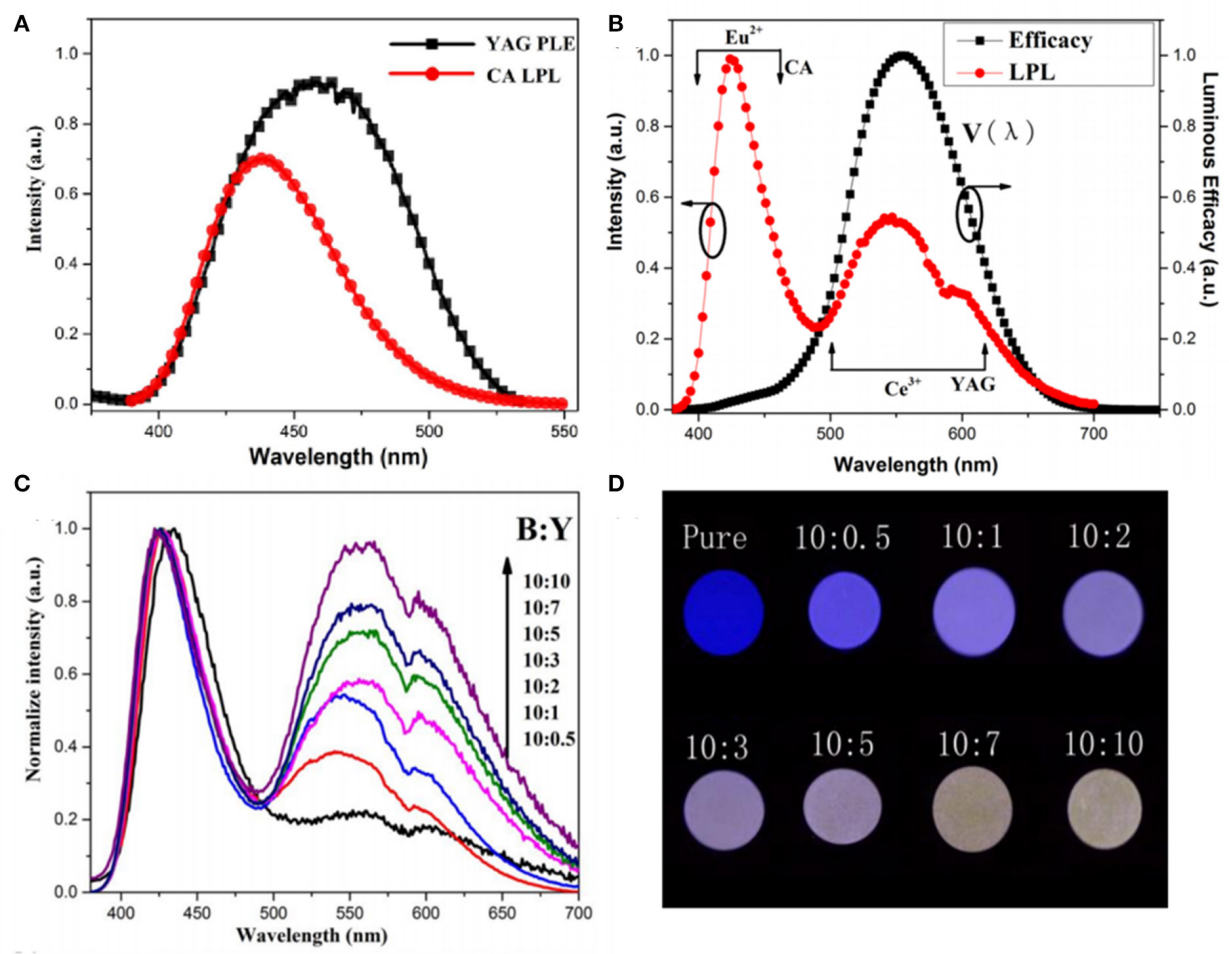
**(A)** The LPL spectrum of CA and the PLE spectrum of YAG. **(B)** The LPL spectrum of composition with B:Y = 10:3 (red line) and the luminous efficiency in photopic vision (black line). **(C)** The normalized LPL spectra of composites with different B:Y weight ratios. **(D)** LPL photographs of composites after exposing to UV lights for 5 min. Reproduced from Chen et al. (2016) with permission from The Royal Society of Chemistry.

In order to overcome the abovementioned problems of LPL color regulation through energy transfer in the same matrix, our group for the first time adopts a method of separating the trap center and luminescence center in two different matrices, utilizing the superior energy storage capacity of CaAl_2_O_4_: Eu^2+^, Nd^3+^ (CA), the high quantum efficiency of Y_3_Al_5_O_12_: Ce^3+^ (YAG), and the great overlap of the LPL spectrum of CA and the PLE spectrum of YAG, to construct CA/YAG composites, as shown in Figure 7 (Chen et al., 2016). The radiation energy transfer from CA to YAG achieves the LPL color adjustable characteristics from blue to white, prolongs the LPL duration time of CA/YAG composite with B:Y = 10:10–48 h compared with 19 h of CA, and heightens the initial LPL brightness to 3,200 mcd/m^2^, which is 2.7 times higher than pure CA. The main reason for the dramatic improvement of LPL performances of CA/YAG composite is that the human visual efficiency of yellow light is much higher than that of blue light although the energy transfer efficiency reduces the overall radiation. The white LPL performances achieved by this method overcame the problem through composite tricolor LPL materials, which required similar decay characteristics to ensure color uniformity in the LPL decay process.

### Adjusting the Occupancy Rate of the Luminescent Center in Multi-Sites

**Figure 8:**
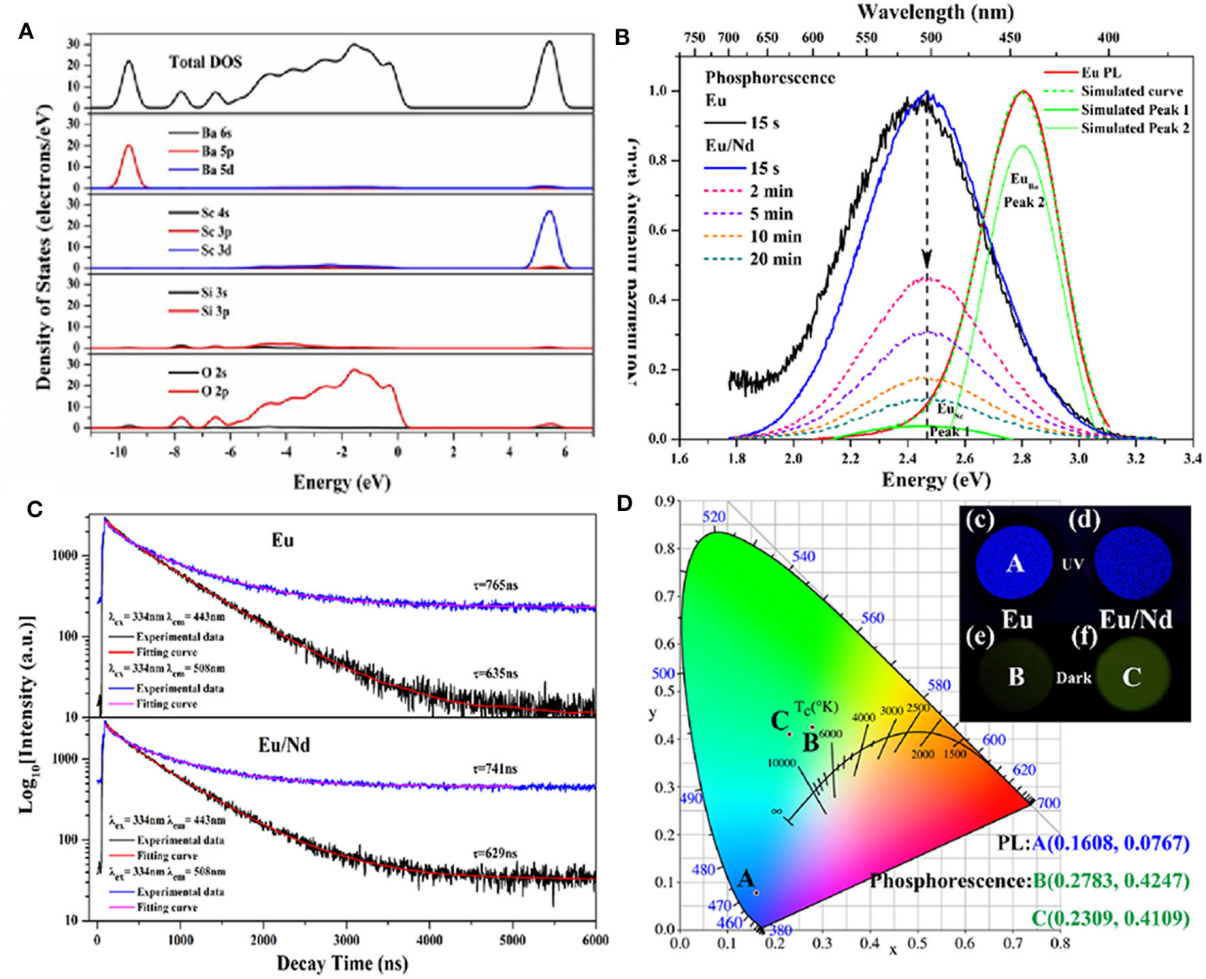
**(A)** Total and partial density of states of BaSc_2_Si_3_O_10_. **(B)** The normalized PL spectrum and LPL spectra of BaSc_2_Si_3_O_10_: 0.01Eu^2+^ and BaSc_2_Si_3_O_10_: 0.01Eu^2+^, 0.01Nd^3+^. **(C)** The luminescence decay curves of BaSc_2_Si_3_O_10_: 0.01Eu^2+^ and BaSc_2_Si_3_O_10_: 0.01Eu^2+^, 0.01Nd^3+^ excited at 334 nm and monitored at 443 and 508 nm, respectively. **(D)** CIE chromaticity coordinates and photographs for the PL and LPL of BaSc_2_Si_3_O_10_: 0.01Eu^2+^ and BaSc_2_Si_3_O_10_: 0.01Eu^2+^, 0.01Nd^3+^. Reproduced with permission from Li G. et al. (2017), copyright 2017, Elsevier.

**Figure 9:**
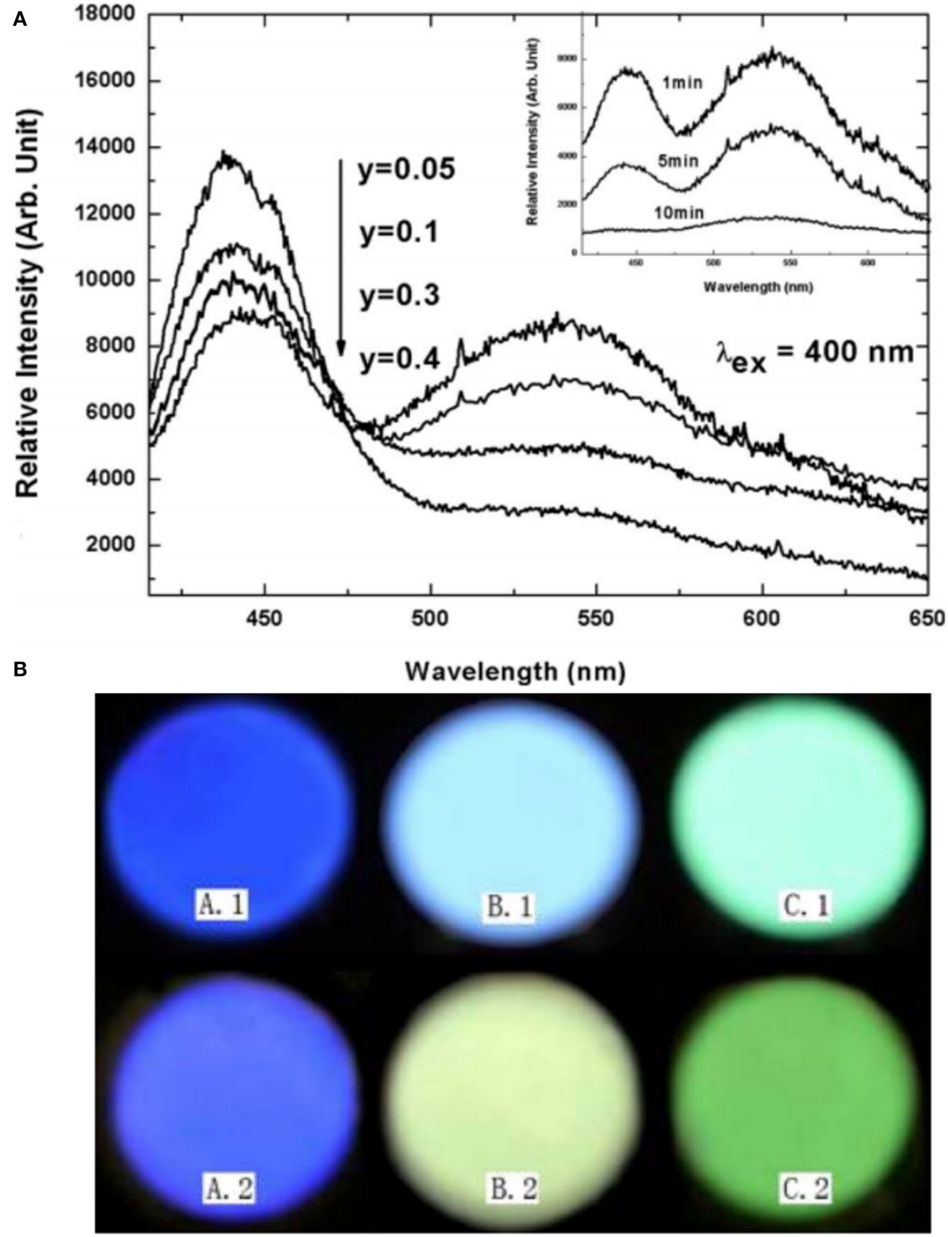
**(A)** PL spectra of Ca_1−3x−y_Al_2_O_4_: xEu^2+^, 2xNd^3+^, ySr^2+^ (*x* = 0.00125, 0.05 ≤ y ≤ 0.4), and the inset shows the LPL spectra of the sample (*y* = 0.4) measured at different times (1, 5, and 10 min). **(B)** Photos of PL (above) and LPL (below). A: CaAl_2_O_4_: xEu^2+^, 2xNd^3+^ (*x* = 0.00125); B: Ca_1−3x−y_Al_2_O_4_: xEu^2+^, 2xNd^3+^, ySr^2+^ (*x* = 0.00125, *y* = 0.4); C: SrAl_2_O_4_: xEu^2+^, 2xNd^3+^ (*x* = 0.00125). Reproduced with permission from Xu et al. (2009b), copyright 2009, IOP Publishing.

In addition to the emergency lighting mentioned above, LPL materials have gradually shown more excellent performance in anti-counterfeiting and other aspects (Jiang et al., 2016; Sandhyarani et al., 2017). At present, the relatively mature fluorescent anti-counterfeiting is to realize visible light output and achieve anti-counterfeiting recognition using ultraviolet excitation. Compared with fluorescent anti-counterfeiting, the LPL anti-counterfeiting is still sustainable after turning off the excitation light source. On this basis, if the color of LPL after turning off the light source is obviously different from that of PL excited by the light source, it will greatly improve the level and force of anti-counterfeiting and provide a more effective guarantee for the safety and reliability of commodities, bills, etc. Here, we achieved this purpose mainly by regulating the Eu^2+^ ions occupation rate of multiple sites in the matrix. Figure 8A presents the total and partial density of states of BaSc_2_Si_3_O_10_ calculated according to the density function theory (Li G. et al., 2017). It can be seen that the valence band (VB) is derived from 2p electronic state of O and the CB is mainly composed of 3d electronic state of Sc. Figure 8B displays that the PL spectrum of BaSc_2_Si_3_O_10_: 0.01Eu^2+^ exhibits a wide asymmetric blue emission band peaking at 443 nm, which can be fitted well into two Gaussian peaks at 443 nm and 508 nm, ascribed to the 5d-4f transitions of Eu^2+^ ion occupying Ba and Sc sites, respectively. As shown in Figures 8C,D, the luminescence decay curves of BaSc_2_Si_3_O_10_: 0.01Eu^2+^ and BaSc_2_Si_3_O_10_: 0.01Eu^2+^, 0.01Nd^3+^ prove that the green LPL at 508 nm is due to Eu^2+^ emission, not some defect level. Based on the above analysis of the electronic structure and luminescence performances, it is believed that only Eu^2+^ ions occupying the Sc sites participate in the LPL process, while Eu^2+^ ions occupying the Ba sites are mainly involved in PL, producing an interesting phenomenon of blue PL and green LPL. Analogously, in CaAl_2_O_4_: Eu^2+^, Nd^3+^ LPL material, the green emission peak at 550 nm was enhanced while the blue emission peak at 440 nm was weakened by the partial replacement of Ca^2+^ by Sr^2+^ ions, as shown in Figure 9 (Xu et al., 2009b). When the doping concentration of Sr^2+^ ions is 0.4 mol, the two emission peaks make the PL color appear as white light. When the excitation light source is removed, the LPL color is yellowish-green on account of the different decay characteristics of Eu^2+^ ions occupying different lattice sites.

## Summary and Prospect

In this review, we provided the development of LPL materials with wavelengths in the 400–600 nm range, mainly introduced our group's research on developing ultra LPL materials based on Eu^2+^ ions, achieved a multicolor LPL phenomenon through energy transfer, and realized the distinct color of PL and LPL performances through adjusting the Eu^2+^ ions occupancy rate to improve the anti-counterfeiting level. However, there are still many areas for further research and development, including but not limited to the following: (a) unearthing novel host lattices and activator ions; (b) controlling excitation and emission wavelengths; (c) structuring a unified test standard; and (d) opening up novel multifunctional application fields.

## Author Contributions

YW was responsible for the framework design, thinking arrangement, and text calibration of the paper. HG was responsible for the paper writing and the investigation of research progress at home and abroad. Both authors contributed to the article and approved the submitted version.

## Conflict of Interest

The authors declare that the research was conducted in the absence of any commercial or financial relationships that could be construed as a potential conflict of interest.
